# Water-Insoluble Polysaccharide Extracted from *Poria cocos* Alleviates Antibiotic-Associated Diarrhea Based on Regulating the Gut Microbiota in Mice

**DOI:** 10.3390/foods12163080

**Published:** 2023-08-16

**Authors:** Yong Lai, Huiling Deng, Qi Fang, Linhua Ma, Hui Lei, Xiurong Guo, Ya Chen, Can Song

**Affiliations:** 1 School of Pharmacy, Southwest Medical University, Luzhou 646000, China; yonglai3210@163.com (Y.L.); fq15510110160379@163.com (Q.F.); 15181428034@163.com (L.M.); huilei@swmu.edu.cn (H.L.); xiurongguo@swmu.edu.cn (X.G.); 2 Chongqing Academy of Science and Technology, Chongqing 401121, China; 13618346171@163.com (H.D.); cq_chenya@163.com (Y.C.); 3 Key Laboratory of Condiment Supervision Technology for State Market Regulation, Chongqing Institute for Food and Drug Administration, Chongqing 401121, China

**Keywords:** *Poria cocos*, water-insoluble polysaccharides, antibiotic-associated diarrhea, gut microbiota, short chain fatty acids

## Abstract

Antibiotics are very effective in treating a variety of bacterial infections, while clinical overuse of antibiotics can lead to diseases such as antibiotic-associated diarrhea. Numerous studies have shown that natural polysaccharides can be used as prebiotics to alleviate antibiotic-associated diarrhea (AAD). *Poria cocos* is a medicinal and edible mushroom widely used for thousands of years in China, and our former study demonstrated that water-insoluble polysaccharide (PCY) has the potential prebiotic function. Therefore, we simulated the digestion and fermentation of PCY using feces from volunteers, and then administered it to C57BL/6 mice with AAD to study its effects on the gut microbiota and metabolites. The results indicated that PCY effectively alleviated the symptoms of AAD in mice, restored the intestinal barrier function, improved the content of short-chain fatty acids (SCFAs), decreased the level of inflammatory cytokines, and changed the structure of gut microbiota by increasing the relative abundance of norank_f__*Muribaculaceae* and unclassified_f__*Lachnospiraceae*, and decreasing that of *Escherichia-Shigella, Staphylococcus* and *Acinetobacter*. This study further demonstrated that PCY is an effective functional prebiotic for improving AAD disease, and provided a new avenue and insight for developing PCY as a functional food or prebiotic for alleviating gastrointestinal diseases.

## 1. Introduction

The antibiotics are widely used as an effective drug to treat a wide range of bacterial infections in hospitals [1]. However, prolonged and inappropriate clinical use of antibiotics increases the risk of a number of complications, and one of the most common complications is antibiotic-associated diarrhea [2]. Normal gut microbiota plays a crucial role in human health [3], and many chronic diseases such as hypertension, diabetes, and inflammatory bowel disease are closely connected to the changes of gut microbiota [4]. Therefore, how to maintain the balance of intestinal microorganisms and reverse antibiotic-induced disorders of gut microbiota are inevitable hot topics in clinical practice.

Numerous studies have shown that the use of polysaccharide prebiotic can regulate gut microbiota and promote the structural integrity of intestinal tissues, thereby restoring the gut imbalance caused by AAD [5,6]. Polysaccharides are a class of long-chain carbohydrates that are widely found in plants and microorganisms [5,7]. Many studies have revealed that polysaccharides of fungal origin have anti-inflammatory, antioxidant, antitumor, antiviral, immunomodulatory and hypoglycemic abilities [8,9]. The intake of polysaccharides is crucial for maintaining intestinal health in human diets, and they are often divided into water-soluble polysaccharides and insoluble polysaccharides based on their water solubility [10,11]. *P. cocos* is a valuable and ancient traditional Chinese medicine, its main active ingredients contain water-soluble polysaccharides, water-insoluble polysaccharides, triterpenoids [12,13,14]. Modern pharmacological studies have shown that polysaccharides extracted from *P. cocos* have good biological activities such as anti-tumor, immunomodulation, hypoglycemia, lipid regulation and liver protection [15,16,17]. Our group previously studied the effect of three main active ingredients from *P. cocos* on mice and found that PCY can increase the number of *Lactobacillus* to regulate the gut microbiota, which is beneficial to the health of the organism [18]. Fungal polysaccharides from medical and edible mushroom sources have great prebiotic potential and are expected to be used to alleviate antibiotic-associated diarrhea, which deserves to be explored in depth.

In this study, we investigated prebiotic effects of water-insoluble polysaccharides of *P. cocos* origin. Firstly, we perform digestion and fermentation experiments of PCY to simulate its effects on healthy people. In addition, we evaluated the alterations and effects of PCY on mice with AAD, and explored its pharmacological mechanisms for regulating gut microbiota to alleviate antibiotic-associated diarrhea. We aimed to investigate the prebiotic properties of PCY for the alleviation of AAD, and to provide new ideas to address the clinical problem of antibiotic-induced intestinal diseases.

## 2. Materials and Methods

### 2.1. Reagents and Chemicals

*P. cocos* were purchased from Sichuan Xin RenTai Pharmaceutical Co., Ltd., Pengzhou, China. Standard monosaccharides were purchased from Shanghai Yuanye Bio-Technology Company, Shanghai, China. Standard SCFAs were purchased from SIGMA Company, Shanghai, China. Reducing sugar content (RSC) assay kit and inflammatory cytokine kit including TNF-α, IL-6, and IL-1β, were purchased from Beijing Solarbio Science Technology Co., Ltd., Beijing, China. Total Antioxidant Capacity Assay (TAOC) Kit with a rapid ABTS method was purchased from Beyotime biotechnology Co., Ltd., Nantong, China.

### 2.2. Extraction and Characteristics of PCY

The extraction and purification of PCY was performed according to a specific method that refers to our previous study [18]. The PMP (1-phenyl3-methyl-5-pyrazolone) pre-column derivatization-HPLC method was applied to analyze the monosaccharides of PCY, and the structure was applied scanning electron microscope (SEM) to observe.

### 2.3. In Vitro Simulated Digestion and Fermentation Experiments of PCY

In vitro simulated digestion and fecal fermentation experiments of PCY were carried out referenced to our team’s previous study [19]. All the saliva, intestinal fluid, gastric fluid and fermentation media used in the experiment were prepared according to previous studies [20,21,22]. In the first part of the simulated digestion experiments, we determined data on residual ash weight, total antioxidant capacity and reducing sugar content of after 50 mg of PCY being treated with digestive solution. Then, after PCY was fermented by the feces of three healthy volunteers, we examined its pH, total antioxidant capacity and reducing sugar content, SCFAs concentration, and effect on the gut microbiota.

### 2.4. Animal Experiments

The Ethics Committee of the Animal Experimentation Center of Southwest Medical University implemented and approved all animal experimentation procedures (license number P20220707-01). All C57BL/6 mice were acclimatized for 1 week in a standard environment with 12-h light-dark cycles, a temperature of 22 °C, and 50% humidity. Twenty-eight C57BL/6 mice were randomly divided into four groups of seven mice each, namely normal control group (NC group), antibiotic-associated diarrhea group (AAD group), PCY treatment group (PCY group), and natural recovery group (NR group). The whole animal experiment was divided into two phases: pathological model phase (1–3d) and experimental recovery period (4–10d), except for the NC group for healthy blank control group only gavage saline per day, the rest of the group were pathological animal model group, during the pathological model period gavage 3 g/kg lincomycin hydrochloride twice a day [23]. In the experimental recovery period, mice of PCY group were gavaged with 300 mg/kg polysaccharides twice a day for 7 d, and we continuously recorded the scores of mouse weight, mental and diarrheal status, and finally collected mouse feces, blood, and cecum tissues and stored in −80 °C refrigerators for subsequent experimental determination.

### 2.5. Histological Examination of the Cecum

We fixed 4 groups of mouse cecum tissues in 10% paraformaldehyde, dehydrated them using anhydrous ethanol, and fixed them with paraffin embedding. The paraffin was cut into slice, and it was stained with hematoxylin for 10 min and washed with tap water. The above section was then differentiated using hydrochloric acid alcohol for 10 s and washed in warm water for 3 min until the color of the nuclei returned to blue. Next it was placed in 85% alcohol for 3 min, the sections were stained with eosin for 3 min and washed with tap water for 5 s, and finally the histological morphology and structure were observed under a light microscope.

### 2.6. 16S rRNA Sequencing

16S rRNA sequencing was carried in Meiji Biotechnology Co., Ltd., (Shanghai, China) using the Illumina platform. The detailed process and specific methods of high-throughput sequencing refer to our previous research [18]. Raw sequence data for fermentation and animal experiment microbiota are saved in the NCBI Sequence Read Archive (SRA) under registration number PRJNA932548 and PRJNA917814.

### 2.7. Detection of SCFAs

LC-MS (Liquid chromatography-mass spectrometry) was used to determine the concentration of SCFAs in mouse fecal samples. According to the SCFAs standard map, the library was retrieved by the data processing system of the chromatography workstation, the composition of SCFAs was confirmed with reference to relevant literature, and the concentration of SCFAs was calculated.

### 2.8. Cytokine Measurement

Enzyme-linked immunosorbent assay (ELISA) is an efficient detection method that uses specific binding of antigen and antibody to carry out immune reaction. The levels of inflammatory cytokines tumor necrosis factor alpha (TNF-α), interleukin 6 (IL-6), and interleukin 1β (IL-1β) were determined in mouse serum by using commercial ELISA kits.

### 2.9. Statistical Analysis

Significance of differences between groups was analyzed using GraphPad Prism 7.04 and SPSS statistical software 19.0. The hierarchical clustering analysis was performed with a Bray-Curtis distance matrix based on ASV level; PCoA was tested using ANOSIM with a Bray-Curtis distance matrix; the method used for the multi-group comparative analysis of microbiota was the Kruskal-Wallis H test with a FDR correction; the method used for the two-group comparative analysis of microbiota was the Wilcoxon rank- sum test with a two-tailed test and FDR correction; LEfSe analyses were performed using the multiple comparison strategy all-against-all (more strict). 

## 3. Results

### 3.1. Characteristics of PCY 

As seen in Figure S1, the monosaccharide composition of PCY determined is glucose. The results of scanning electron microscopy are in Figure S2, PCY exhibit a flaky or crumbly stacked morphology with smooth surface morphology, and it has a complete and uniform structure, which is uniformly smooth and tightly packed at both 30 μm and 100 μm magnifications.

### 3.2. In Vitro Simulated Digestion of the PCY 

**Figure 1:**
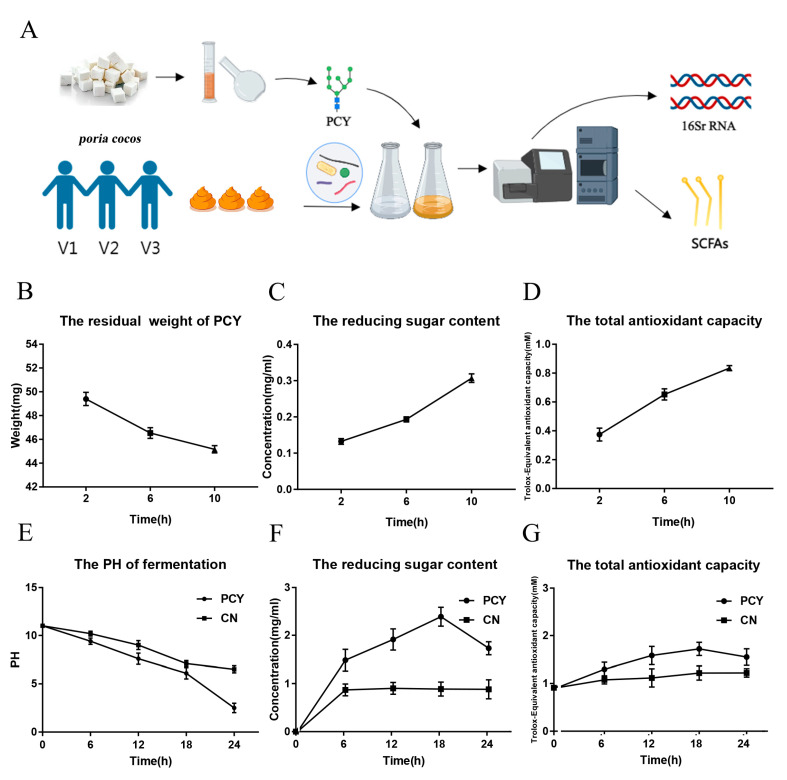
(**A**) Schematic diagram of simulated PCY fermentation in vitro. (**B**) The residual ash weight of PCY in digestion. (**C**) The RSC of PCY in digestion. (**D**) The TAOC of PCY in digestion. (**E**) The pH of PCY in fermentation. (**F**) The RSC of PCY in fermentation. (**G**) The TAOC of PCY in fermentation.

The whole digestive process includes three stages: saliva digestion, gastric juice digestion and intestinal juice digestion, and the environmental pH value of each stage is different. After 50 mg of water-insoluble polysaccharides entered the simulated digestion stage, the residual mass of polysaccharides gradually decreased, and the measured weight was 45.1 + 1.2 mg at 10 h (Figure 1B). As shown in Figure 1C, reducing sugars mainly include glucose, and a small part of water-insoluble polysaccharides is decomposed into reducing sugars under the action of saliva, gastric juice and intestinal juice. The results show that the RSC gradually increases with time, and the RSC is 0.295 + 0.018 mg/mL when the simulated digestion is completed. When using Trolox as a standard, the TAOC of the sample is expressed as TEAC. As shown in Figure 1D, the TAOC of PCY gradually increased during the simulated digestion, and the TAOC of polysaccharide digestion was 0.829 + 0.08 mM at 10 h.

### 3.3. In Vitro Fermentation of PCY

**Figure 2:**
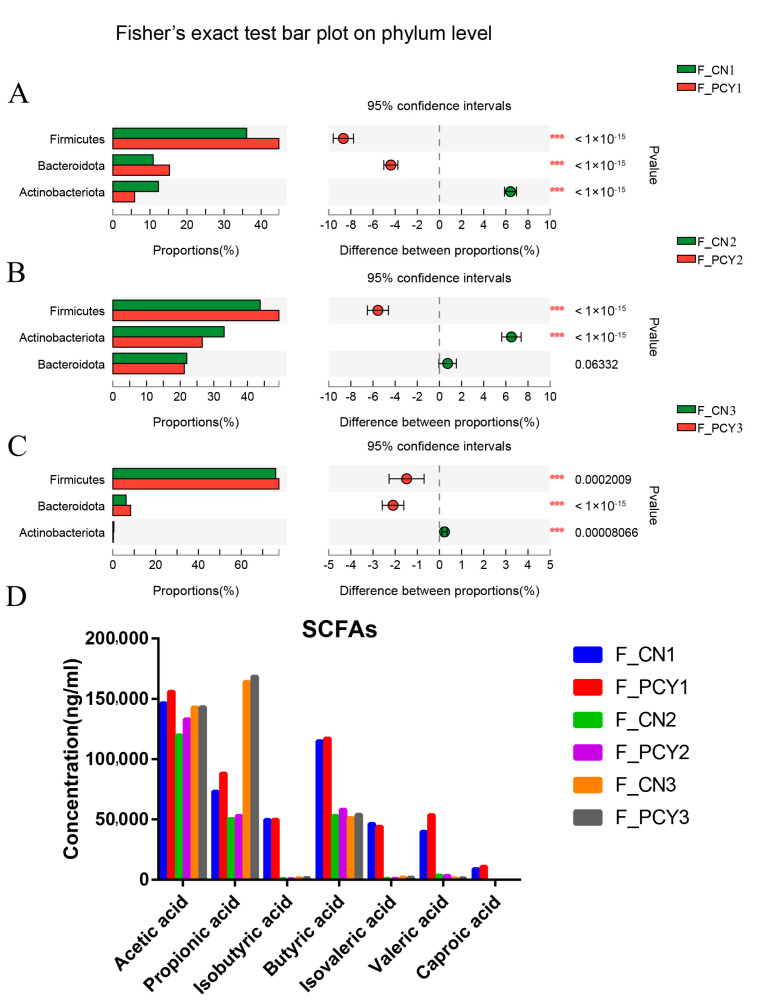
(**A**) Difference between F_CN1 and F_PCY1 at the phylum level. (**B**) Difference between F_CN2 and F_PCY2 at the phylum level. (**C**) Difference between F_CN3 and F_PCY3 at the phylum level. (**D**) The changes of SCFAs in all groups. *** *p* < 0.001.

A schematic diagram of healthy human fecal fermentation for PCY is shown in Figure 1A. The changes of pH, RSC, and TAOC during the fermentation of human feces were determined (Figure 1E–G). The pH of the fermentation broth decreased with time, while the RSC and TAOC increased with time. The results of microbiota analysis of PCY after 24 h fermentation in healthy human feces and the results of its metabolites are shown in Figure 2. The results of microbial composition of fresh feces from healthy volunteers are shown in the supplementary material (Figure S3), and the normal human gut microbes at the phylum level contain mainly Firmicutes, Bacteroidota, Proteobacteria, Actinobacteriota, and Fusobacteriota. At the end of fermentation, in the fisher’s exact test bar plot of the PCY and NC groups, the relative abundance of Firmicutes and Bacteroidota significantly increased, while the relative abundance of Actinobacteriota significantly decreased on phylum level (Figure 2A–C). The results of SCFAs in the PCY and NC groups are shown in the Figure 2D, and the concentration of propionic and acetic acid in the PCY group was higher, while the concentration of acetic, propionic, butyric, valeric, and caproic acid have no significant difference. 

### 3.4. General Data of C57BL/6 Mice

**Figure 3:**
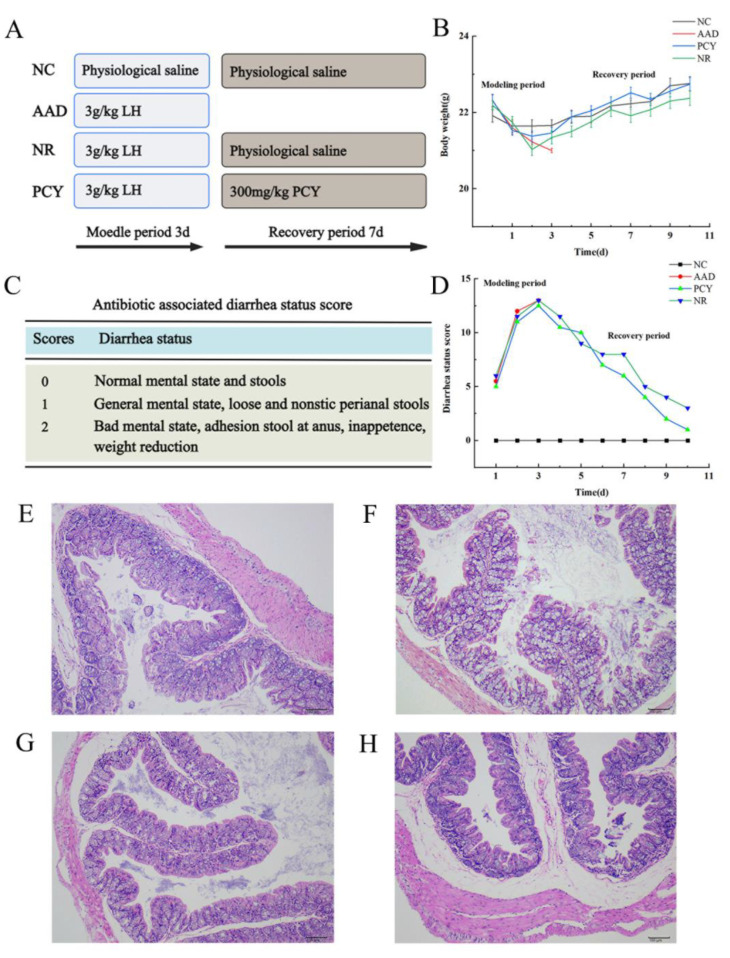
(**A**) Schematic diagram in the animal experiment. (**B**) Changes of body weight in mice. (**C**) Diarrhea status scoring methods. (**D**) Changes of diarrhea status score in mice. (**E**) Histological changes in the cecum of the NC group. (**F**) Histological changes in the cecum of the AAD group. (**G**) Histological changes in the cecum of the PCY group. (**H**) Histological changes in the cecum of the NR group.

The whole animal experiment lasted 10 days, which was divided into a 3-daypathological model modeling period and a 7-day experimental treatment period (Figure 3A). As shown in Figure 3B, mice’s weight dropped rapidly after antibiotics during the model period, and then the PCY group of mice received polysaccharide treatment and recovered their weight better compared to the NR group. Moreover, the status scale for antibiotic-associated diarrhea in C57BL/6 mice is shown in Figure 3C, the diarrhea status score of mice in the PCY group significantly decreased than that in the NR group during the monitoring of feeding (Figure 3D). 

### 3.5. Effect of PCY on Cecal Tissues

After HE staining and photographing, Figure 3 exhibited the histopathological changes of the cecum in C57BL/6 mice. The histological features of the blank control group are shown in Figure 3E, with intact intestinal mucosa and regular arrangement of epithelial cells. The cecal tissues of mice in the AAD group showed severe histopathological changes, such as mucosal defects, massive inflammatory cells infiltration, edema, and incomplete goblet cells (Figure 3F). After a one-week recovery period, the mice with the administration of PCY has the best effect in the histological characteristics of the cecum (Figure 3G). Compared with mice in the NR group, PCY significantly improved the defects of the intestinal mucosa, reduced the number of inflammatory cells, and reduced the edema of the submucosa (Figure 3H). It has been shown that the intestinal mucosa is defective in the intestinal tissue of AAD hosts, and there are pathological features such as inflammatory cell infiltration and edema, which are consistent with the characteristics of the mice in the AAD group in this study.

### 3.6. Composition and Diversity of the Gut Microbiota

The feces of C57BL/6 mice were used for 16S rRNA in the experiment. The PCR amplification results of feces in the AAD group are shown in the supplementary material (Figure S4), and the serious damage caused by antibiotics to the gut microbiota fully demonstrates that our experiments have successfully created a pathological model, which is consistent with the previous study [24]. The Rarefaction curve is used to determine whether the amount of sequencing data for the gut microbiota of each sample is reasonable, and the results show that at the current sequencing depth, each sample can be used for further analysis (Figure S5). 

**Figure 4:**
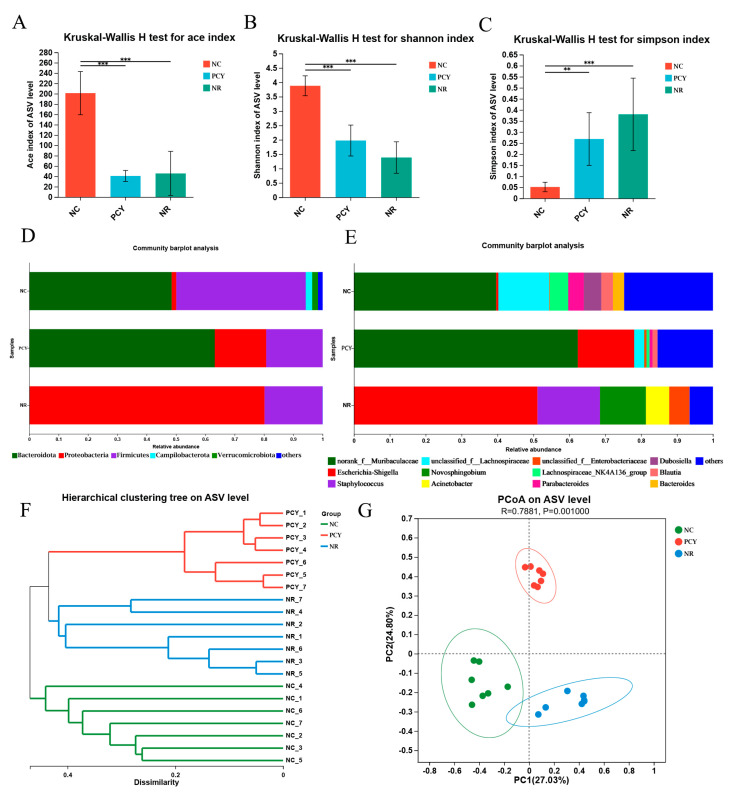
(**A**) Ace index comparation. (**B**) Shannon index comparation. (**C**) Simpson index comparation. (**D**) Difference of three groups at the phylum level. (**E**) Difference of three groups at the genus level. (**F**) Hierarchical clustering tree on ASV level of mice in three groups. (**G**) PCoA on ASV level of mice in three groups. ** *p* < 0.01, *** *p* < 0.001.

α-diversity contains the Simpson index, Ace index, and Shannon index (Table S1). As shown in Figure 4A–C, the types and numbers of gut microorganisms in the AAD and NR groups were significantly lower than those in the NC group, and the differences were statistically significant. Results of α-diversity demonstrated that the microbial communities of the experimental mice were severely damaged by lincomycin hydrochloride, and the application of PCY improved the microbial diversity of the mouse gut microbiota compared to naturally recovered mice. Firmicutes and Bacteroides are the two dominant phylum for maintaining gut microbiota balance at the phylum level. As demonstrated in Figure 4D, there are Bacteroidota, Proteobacteria, Firmicutes, Campilobacterota, and Verrucomicrobiota on mice gut microbiota in NC group. The relative abundance of Proteobacteria in the NR group surprisingly accounted for most of the bacterial phylum level, which was significantly higher than that in the NC group, while the abundance of Proteobacteria was significantly reduced after the administration of PCY. The number of *Escherichia-Shigella* accounts for half of the level of gut microbiota genus in the NR group, and it is the most common pathogen in humans (Figure 4E). As shown in Figure S6, the heat map detected the microbial community changes at the genus level, PCY has obviously regulated the genus level of the mouse gut microbial community. 

β-Diversity was used to investigate similarities between groups. PCoA (principal co-ordinates analysis) is a visualization method to study data similarities or differences, showing differences between and within groups by analyzing structural changes in the gut microbiota of mice. As shown in Figure 4G, there are significant differences between the three groups (*p* = 0.001). The intestinal microbiota between the experimental group and the control group showed a clear separation, and the inconsistency with the blank control group without any treatment indicated the successful establishment of the AAD model. In Figure 4F, Hierarchical clustering tree on ASV level demonstrated that the clustering tree branches of the NC and PCY groups are clearly similar, while the NR group differs from them. The above results show that in addition to being able to reverse the damage caused by antibiotics, PCY can also cause better probiotic changes by regulating the microbiota of AAD mice.

### 3.7. Composition Analysis of the Gut Microbiota

**Figure 5:**
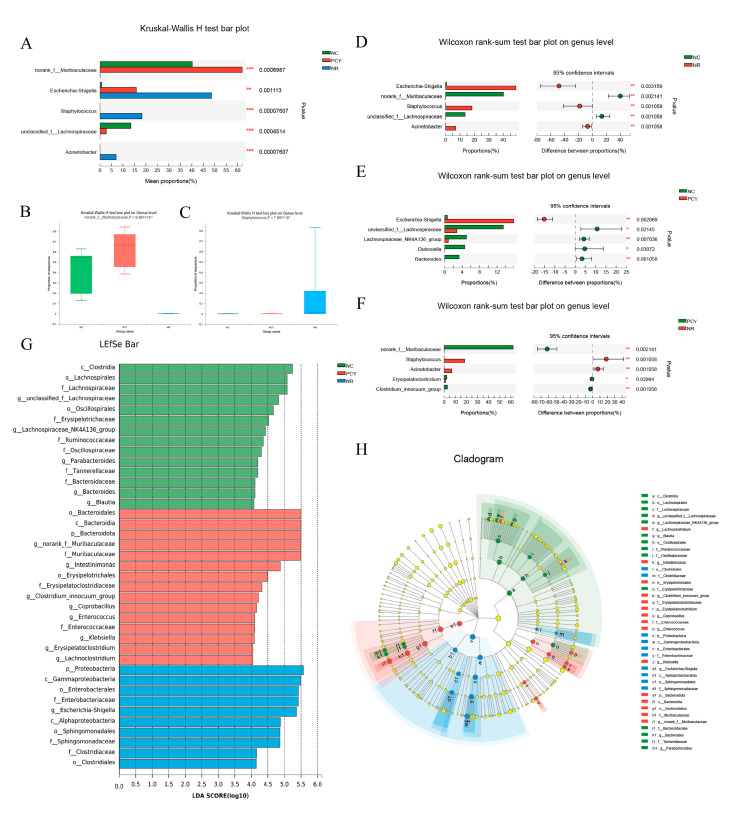
(**A**) Difference of three groups at the genus level. (**B**) Kruskal−Wallis H test box plot of norank_f__*Muribaculaceae*. (**C**) Kruskal−Wallis H test box plot of *Staphylococcus*. (**D**) Difference between NC and NR groups at the genus level. (**E**) Difference between NC and PCY groups at the genus level. (**F**) Difference between PCY and NR groups at the genus level. (**G**) LEfSe analysis. (**H**) Taxonomic cladogram of LEfSe. * *p* < 0.05, ** *p* < 0.01, *** *p* < 0.001.

The comparative analysis of the three groups of gut microbiota at the genus level are presented by Kruskal-Wallis H test bar plot and Wilcoxon rank-sum test bar plot. As demonstrated in Figure 5A, PCY increased the relative abundance of norank_f__*Muribaculaceae, Erysipelatoclostridium* and *Clostridium_innocuum*_group and decreased the relative abundance of *Staphylococcus* and *Acinetobacter* compared to the NR group. The Kruskal-Wallis H test box plot of norank_f__*Muribaculaceae* and *Staphylococcus* in three groups were seen in Figure 5B,C, there are significant difference on norank_f__*Muribaculaceae* and *Staphylococcus* between groups. In the comparison of NC and NR groups, the relative abundance of *Escherichia-Shigella, Staphylococcus* and *Acinetobacter* increased in the NR group, while the relative abundance of norank_f__*Muribaculaceae* and unclassified_f__*Lachnospiraceae* decreased (Figure 5D). As seen in Figure 5E, the use of antibiotics increased the relative abundance of *Escherichia-Shigella* and decreased the relative abundance of unclassified_f__*Lachnospiraceae, Lachnospiraceae*_NK4A136_group, *Dubosiella* and *Bacteroides* compared to the NC group. As shown in Figure 5F, PCY significantly increased the relative abundance of norank_f__*Muribaculaceae* compared to the NR group (*p* < 0.01), while the beneficial bacteria were almost absent in the naturally recovery group. The results of linear discriminant analysis effect size (LEfSe) are shown in histogram of LDA value distribution and branching of species evolution (Figure 5G,H). We compared high-dimensional categories and explored differences in bacterial community dominance. There were 14 significantly different species in the NC group, the PCY group had 15 significantly different species in abundance, significant bacteria were *Muribaculaceae* and *Lachnoclostridium*, while the NR group had only 10 significantly different species with many harmful bacteria. 

**Figure 6:**
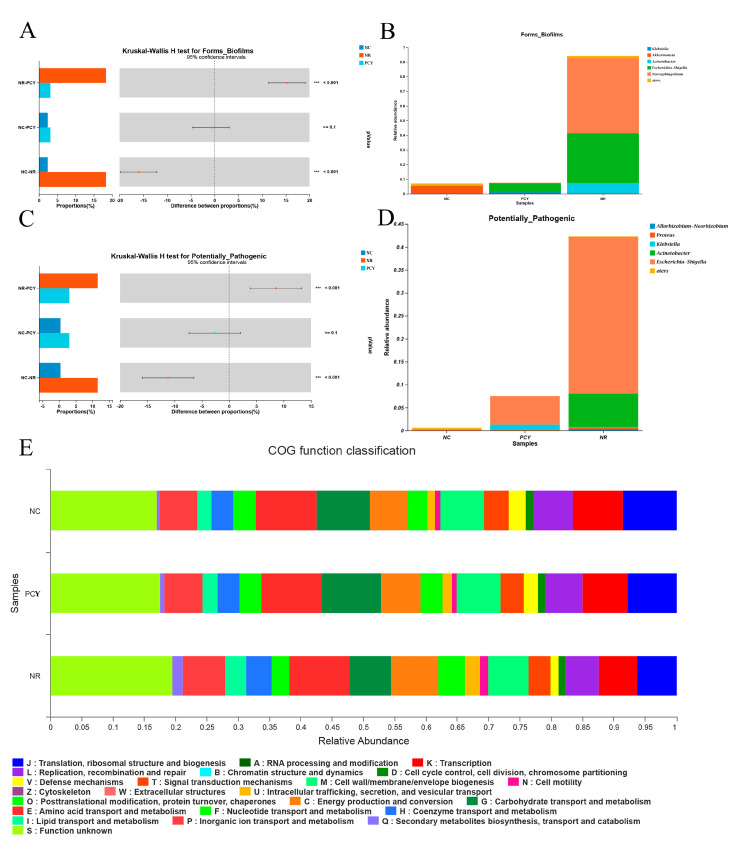
(**A**) Forms of Biofilms comparation. (**B**) The relative abundance of Biofilms in three groups. (**C**) Potentially Pathogenic comparation. (**D**) The relative abundance of Potentially Pathogenic in three groups. (**E**) COG function classification. *** *p* < 0.001.

The biofilm and potential pathogenicity predicting of the three groups microbiota were evaluated using the Kruskal-Wallis H test method, as shown in Figure 6A–D, microbiota in the NC and PCY groups had less biofilm and potentially pathogenic bacteria compared to the NR group. COG (Clusters of Orthologous Groups) is a method of differential gene function annotation and has a prominent role in predicting the function of proteins [25]. The COG function classifications are shown in Figure 6E and Figure S7. In addition, the correlation heat map of protein function prediction for the three groups is shown in Figure S8.

### 3.8. Effect of PCY on SCFAs

**Figure 7:**
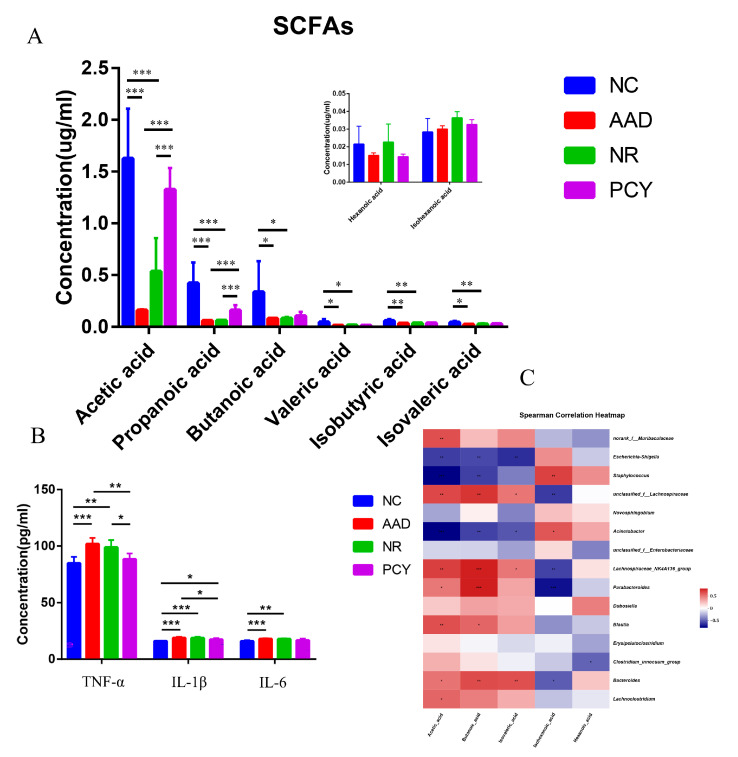
(**A**) Changes in concentrations of SCFAs. (**B**) Changes in the secretion of inflammatory cytokines. (**C**) Spearman Correlation Heatmap of bacteria and SCFAs. * *p* < 0.05, ** *p* < 0.01, *** *p* < 0.001.

The results of SCFAs content in the mouse feces in each group are shown in Figure 7A. The concentration of acetic acid and propionic acid significantly decreased in the AAD group than the NC group (*p* < 0.001). The use of lincomycin hydrochloride decreased SCFAs in mice compared to healthy mice, probably because the antibiotic suppressed SCFA-producing bacteria. The level of acetic acid in the PCY group (1.63 ± 0.48 μg/mg) was significantly higher than that in the NR group (0.54 ± 0.22 μg/mg) and the AAD group (0.15 ± 0.01 μg/mg) (*p* < 0.001). butyric acid in the PCY group was 0.42 ± 0.2 μg/mg, which was statistically higher than that in the NR group (*p* < 0.001). In addition, the concentrations of butyric, valeric, iso-butyric, isovaleric, hexanoic, and iso-hexanoic acid in the PCY group did not change significantly between groups in our experiment. 

The value of environmental factor VIF is shown in Table S2, the strength of the correlation was assessed using the color depth on the heatmap, and the results indicated that acetic, butyric, isovaleric, hexanoic and iso-hexanoic acid are strongly correlated with some bacteria after our filtering (Figure 7C). For example, acetic acid was positively correlated with norank_f__*Muribaculaceae* and unclassified_f__*Lachnospiraceae*; acetic acid was negatively correlated with *Staphylococcus* and *Acinetobacter*.

### 3.9. Effect of PCY on Cytokines

As demonstrated in Figure 7B, the concentrations of TNF-α, IL-6 and IL-1β were significantly higher in the AAD group than they were in the NC group (*p* < 0.001), and the concentrations of TNF-α, IL-6 and IL-1β were also higher in the NR group than they were in the NC group (*p* < 0.01), indicating the success of our pathological model of AAD. PCY significantly decreased the levels of TNF-α and IL-1β compared with the AAD group (*p* < 0.05) and decreased the levels of TNF-α compared with the NR group (*p* < 0.05). The inflammatory cytokine concentration of PCY group mice is more similar to that of healthy mice in NC group, while the inflammatory cytokines in the serum of naturally recovered mice tended to be closer to those of mice in the AAD model group, indicating that the intake of PCY accelerated the recovery of mice to a healthy state.

## 4. Discussion

In recent years, numerous studies have revealed a complex and close connection between various target organs and gut microbiota, including the gut-brain axis, the gut-lung axis, the gut-skin axis, and the gut-liver axis [26,27]. Gut microbiota is in dynamic equilibrium, but is susceptible to a variety of factors such as diet, disease, and antibacterial drugs. The occurrence and development of a series of diseases are inextricably linked to the changes of the gut microbiome, among them, antibiotic-related diarrhea, as the most common type of disease in clinical practice, is directly related to the gut microbiota [28]. A healthy gut contains thousands of microorganisms, among which the balance of commensal bacteria, probiotics and pathogens is very important, and together they play a unique role in regulating the gut microbiota, such as inflammatory signaling, short-chain fatty acids, bile acids and lipid metabolism [29,30]. Some polysaccharides have been reported to be used as prebiotics to improve AAD, cereus sinensis polysaccharide improves AAD symptoms by reversing microbial dysfunction, and Panax ginseng polysaccharides altered the composition and diversity of gut microbiota in mice with AAD to promote recovery of health [24,28]. In this project, mice were able to repair intestinal mucosal integrity and intestinal barrier function better and faster by ingesting *P. cocos* water-insoluble polysaccharides, suggesting that PCY has the potential to be a prebiotic for improving intestinal function.

In our primary simulated digestion, polysaccharides readily form polymers in solution, and the disruption of aggregates of polymer chains and cleavage of glycosidic bonds lead to changes in polysaccharide body weight according to previous study [22,31]. During the experiment, the residual weight of PCY decreased, the RSC gradually increased, and the TAOC gradually increased, suggesting that the theory is consistent with our experimental results [32]. Together with the results of RSC and TAOC, we hypothesized that the disruption of aggregates in water-insoluble polysaccharides and the breakage of some glycosidic bonds led to a slight decrease in the weight of PCY. The results of microbiota analysis in simulated fermentation told us that PCY has the ability to enhance Firmicutes and Bacteroidota, two major bacterial phyla of the gut microbiota contain many probiotic bacteria, such as *Lactobacillus* [33]. Studies have demonstrated that the ratio of Firmicutes to Bacteroidetes abundance is closely connected to the development of obesity, with higher relative abundance of Bacteroidetes being less likely to be obese [34]. In addition, acetic acid is the main metabolite of the fermentation of indigestible polysaccharides and is vital source of energy [35]. Propionic acid is the main product of the phylum Bacteroidetes, which is involved in the regulation of immunity and can inhibit hepatic cholesterol synthesis [36]. The results indicated that the gut microbiota of each volunteer is individual and the results obtained with or without the addition of water insoluble polysaccharides to the samples are different. Consequently, the administration of PCY had effect on the gut microbiota and increased the concentration of SCFAs after the fermentation.

The results of 16Sr RNA high sequencing in animal experiment have demonstrated the strong prebiotic potential of PCY, as evidenced by its ability to increase probiotics and decrease harmful bacteria in mice gut microbiota, alleviating the damage to the intestinal microbiota from antibiotic-associated diarrhea. Researches have indicated that Proteobacteria contains many pathogenic bacteria, such as *Escherichia coli, Helicobacter pylori* and *Salmonella*, which can cause dysbiosis in gut microbiota [37]. Proteobacteria are considered to be one of the pathogenic endotoxin-producing bacteria, and the accumulation of large amounts of endotoxin in the intestine can cause many pathological changes, such as intestinal inflammation, immune disorders and functional damage to the intestinal mucosa [38]. Recent studies have shown that expansion of *Escherichia-Shigella* in gut is strongly associated with the occurrence and development of the disease [39]. Comparison of the three groups at the fecal bacterial phylum and genus level showed that antibiotics cause disturbances in the gut microbiota and that the naturally recovered gut microbiota leads to an undesirable multiplication of Proteobacteria. The administration of PCY can alleviate this process and reduce the number of Proteobacteria bacteria to roughly normal levels. *Muribaculaceae* belong to the classification of the phylum Bacteroidetes and are the main utilizers of mucin monosaccharides [40]. Numerous studies have shown that *Muribaculaceae* play an important role in the energy metabolism of the intestine, are functionally diverse in degrading complex carbohydrates, and are considered to exist as beneficial bacteria [41,42]. Treatment with lincomycin hydrochloride can deplete *Muribaculaceae*, and the number of *Muribaculaceae* cannot be restored to the original number even after stopping antibiotic treatment and waiting for the organism to recover naturally, while PCY can facilitate this process. PCY intake increased the relative abundance of unclassified_f__*Lachnospiraceae* among the three groups (*p* < 0.01). unclassified_f__ *Lachnospiracea* is a potential beneficial bacterium, which has considerable capacity in the utilization of dietary fiber such as polysaccharides, and ferments carbohydrates to produce butyric and acetic acid to provide energy [43]. The increase in the abundance of *Lachnospiraceae* is closely related to the increase of plant diet rich in fiber and short-chain fatty acids. Thus, the upregulation of *Lachnospiraceae* and *Muribaculaceae* may indicated that PCY has the potential to promote the treatment of gastrointestinal diseases. *Escherichia-Shigella, Staphylococcus* and *Acinetobacter* were abundantly present in the NR group, while they were little in the NC and PCY groups with statistically significant differences. This result clearly indicates that PCY has the ability to reduce harmful bacteria, thus creating a near normal intestinal microbial environment. *Acinetobacter* are a group of non-fermentative gram-negative bacilli and *Staphylococcus* are common purulent cocci, both are widely found in nature and are important conditional pathogens and common bacteria causing hospital infections today [44,45]. *Escherichia-Shigella* is normally present in the healthy intestine, but its excess can cause disease, and studies have shown a strong correlation between *Escherichia-Shigella* and the severity of non-alcoholic steatohepatitis disease [46]. Naturally recovered mice produce numerous pathogens in the intestine that hinder the process of restoring health. Interestingly, the PCY treated mice, like the normal group of mice, do not produce these pathogens and increase the number of beneficial bacteria such as norank_f__*Muribaculaceae* and unclassified_f__*Lachnospiraceae*.

SCFAs, as carboxylic acids with less than 6 carbon atoms, are important metabolites of intestinal microorganisms [47]. SCFAs are a key source of energy for intestinal cells and can regulate energy homeostasis and metabolism, and SCFAs can regulate immune response and participate in inflammatory response [48]. SCFAs have also been found to inhibit tumor cell proliferation and promote apoptosis [49]. In our study, antibiotics significantly disrupted the intestinal barrier and affected the structure and function of the intestinal microbial community in AAD mice, leading to a substantial reduction in SCFAs. The damage caused by the drug could not be reversed by the natural recovery of the mouse organism even within one week after the cessation of antibiotics. However, the use of PCY was effective in ameliorating this situation, with significantly higher concentrations of butyric acetate in the major SCFAs compared to mice in the AAD group. PCY, a water-insoluble polysaccharide that is difficult to digest, can be used by bacteria in the intestine to produce SCFAs, so the concentration of SCFAs were increased simultaneously, which indicates that the results of our metabolome are in general agreement with the previous microbiota analysis.

**Figure 8:**
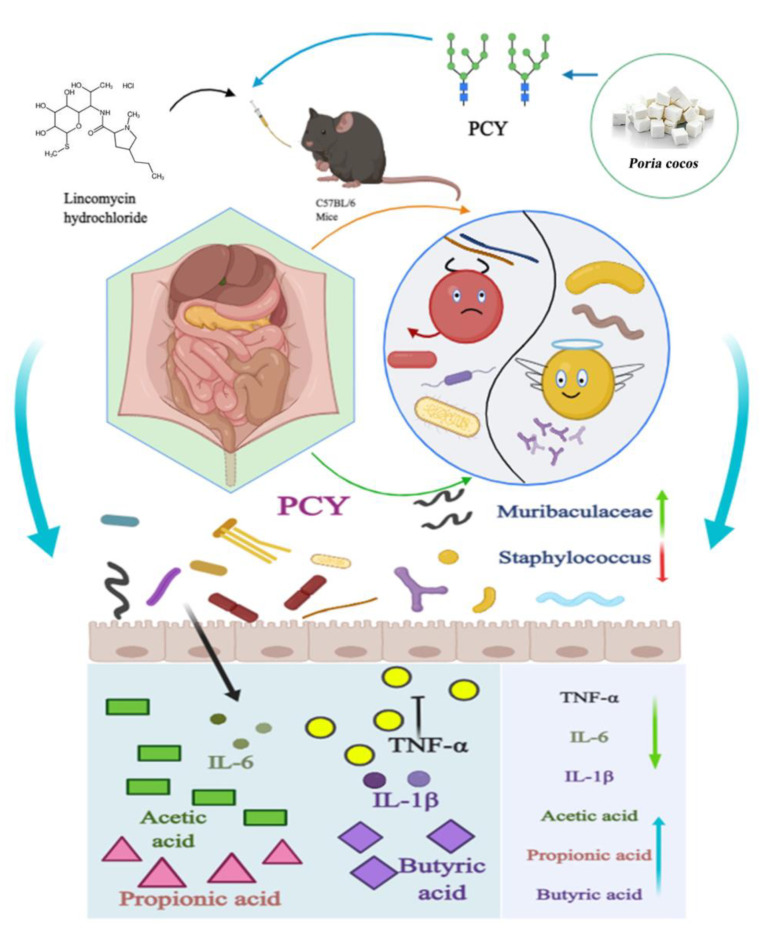
Schematic representation of PCY to alleviate AAD in C57BL/6 mice.

The pathophysiological features of antibiotic-associated diarrhea include an inflammatory response and the release of multiple inflammatory cytokines, and excessive inflammatory cytokines release leads to severe intestinal mucosal damage and dysfunction [50,51]. TNF-α, IL-1β, and IL-6 are the major inflammatory cytokines in the body and play an important role in maintaining gastrointestinal homeostasis [52]. TNF-α is a key promoter of the inflammatory response following intestinal disruption and be able to promote the production and gene expression of IL-1β, and IL-6 [53]. AAD is strongly associated with disruption of intestinal structure, dysbiosis of gut microbiota, inflammatory response and SCFA reduction, as shown in our study, in which mice in the AAD model group had defective and incomplete intestinal mucosa, altered intestinal microbial community structure and composition, reduced beneficial bacteria, increased harmful bacteria, reduced production of SCFAs, and overproduction of inflammatory cytokines. Naturally recovered pathological mice did not perform well in the recovery process, accompanied by the proliferation of some harmful bacteria such as unclassified_f__*Lachnospiraceae* and norank_f__*Muribaculaceae*, which can lead to a large accumulation of harmful substances in the intestine and have a bad effect on improving the condition. Surprisingly, after one week administration of PCY, the mice showed great improvement and recovery, as evidenced by homogeneous and intact intestinal mucosal structure, reduced inflammatory cell infiltration and an overall approach to the intestine of healthy mice, normalized gut microbiota structure and composition, increased relative abundance of probiotics, increased concentration of functionally important SCFAs, and decreased inflammatory cytokines to normal levels. Schematic representation of PCY to alleviate AAD in C57BL/6 mice are in Figure 8. Although our study is not exhaustive and we have not explored the restorative mechanism of a specific protein for AAD, the alteration of gut microbiota, the increase of SCFAs and the decrease of inflammatory cytokines are sufficient to demonstrate the effective alleviating effect of PCY on AAD. Particularly, previous studies on water-soluble polysaccharides have been very detailed, and it is valuable that PCY, as a special water-insoluble polysaccharide, can exert such an excellent effect, which is very important for the in-depth development of Chinese traditional medicine *Poria cocos*. Discovering the new function of water insoluble polysaccharide can realize the secondary utilization of common Chinese medicine, and it is also another novel strategy to find prebiotics to improve gastrointestinal diseases.

## 5. Conclusions

In summary, we first performed digestion simulations and in vitro fermentation experiments to study the properties of PCY. And then we acted PCY on mice with AAD to investigate its effects on the mice’s gut microbiota and metabolites. The results suggest that PCY have beneficial effects on AAD mice. Specifically, PCY facilitated the recovery of intestinal mucosa and barrier function, altered the composition and structure of the gut microbiota, increasing the relative abundance of beneficial bacteria, such as norank_f__*Muribaculaceae* and unclassified_f__*Lachnospiraceae*, and decreasing the relative abundance of harmful bacteria, such as *Escherichia-Shigella, Staphylococcus* and *Acinetobacter*, and increasing the production of important SCFAs, such as acetic acid and propionic acid. In addition, PCY decreased the levels of inflammatory cytokines and inhibited the inflammatory response, thereby better facilitating the recovery of AAD in mice. This study further demonstrates the potential of PCY as a prebiotic, and the results provide a basis for the development of a prebiotic that is effective in alleviating AAD.

## Data Availability

The data presented in this study are available on request from the corresponding author.

## Supplementary Materials

The following supporting information can be downloaded at: https://www.mdpi.com/article/10.3390/foods12163080/s1, Figure S1: The high-performance liquid chromatograms of PCY; Figure S2: The scanning electron micrograph of PCY in 100 μm and 30 μm; Figure S3: The results of microbial composition of fresh feces from healthy volunteers; Figure S4: The PCR amplification results of AAD group mice’s feces; Figure S5: The Rarefaction curve; Table S1: The α-diversity of microbial communities in mice; Figure S6: The heat map of microbial community at the genus level; Figure S7: The COG function classification in three groups; Figure S8: The correlation heat map of protein function prediction for the three groups; Table S2: The value of environmental factor VIF.
