# Supplementary material for: Water-Insoluble Polysaccharide Extracted from Poria cocos Alleviates Antibiotic-Associated Diarrhea Based on Regulating the Gut Microbiota in Mice
Source: Foods. 2023 Aug 16;12(16):3080. doi: 10.3390/foods12163080 (PMC10453245; doi:10.3390/foods12163080)
Supplement: Supplementary file 1 [file foods-12-03080-s001.zip › foods-2492232-supplementary.pdf]

Supplementary Materials:

Water-Insoluble Polysaccharide Extracted from *Poria cocos* Alleviates Antibiotic-Associated Diarrhea Based on Regulating the Gut Microbiota in Mice

Yong Lai [1], Huiling Deng [2,3], Qi Fang [1], Linhua Ma [1], Hui Lei [1], Xiurong Guo [1], Ya Chen [2] and Can Song [1,*]

[1] School of Pharmacy, Southwest Medical University, Luzhou 646000, China
[2] Chongqing Academy of Science and Technology, Chongqing 401121, China
[3] Key Laboratory of Condiment Supervision Technology for State Market Regulation, Chongqing Institute for Food and Drug Administration, Chongqing 401121, China
* Correspondence: cansong@swmu.edu.cn

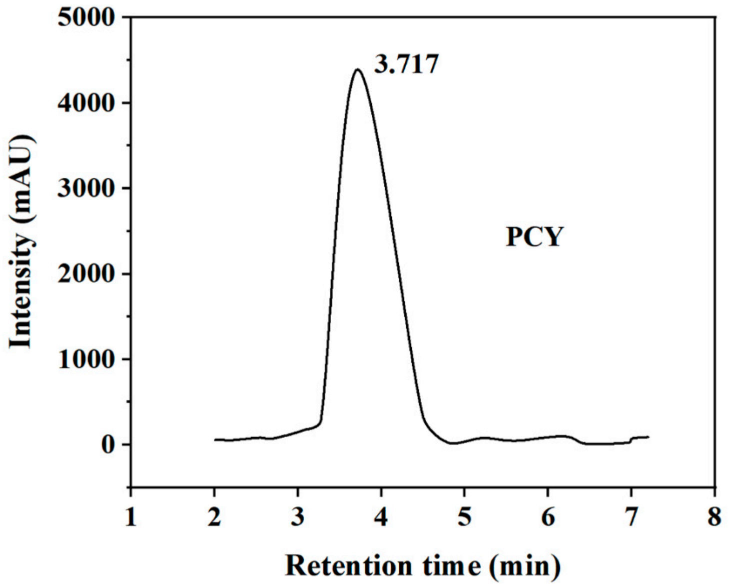

Figure S1. The high-performance liquid chromatograms of PCY.

A
B

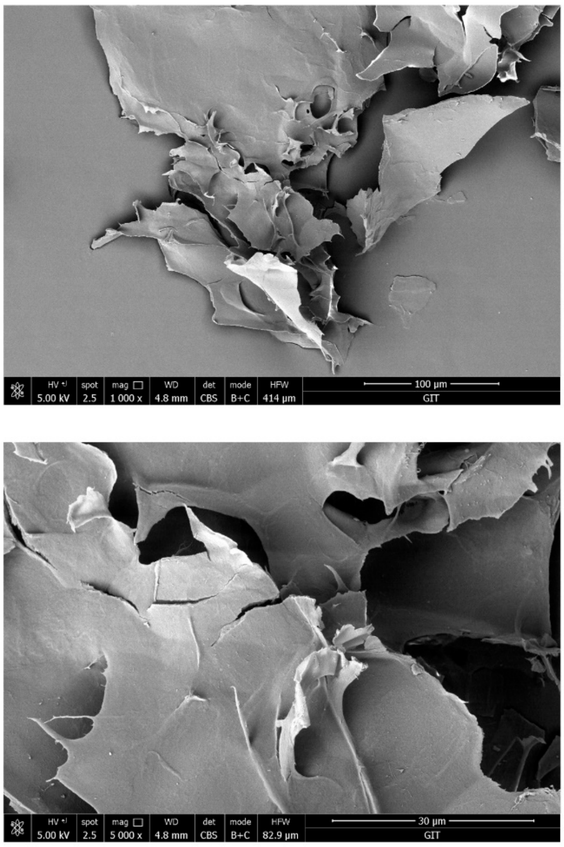

Figure S2. The scanning electron micrograph of PCY in 100um (A) and 30um (B).

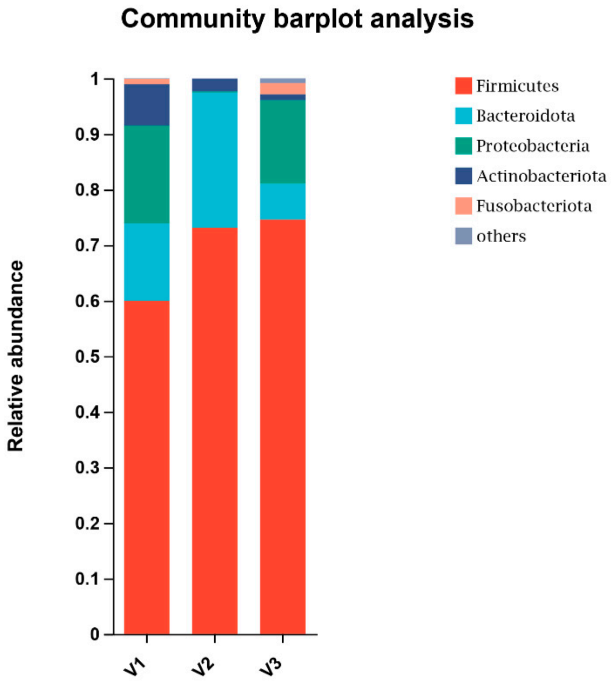

Figure S3. The results of microbial composition of fresh feces from healthy volunteers.

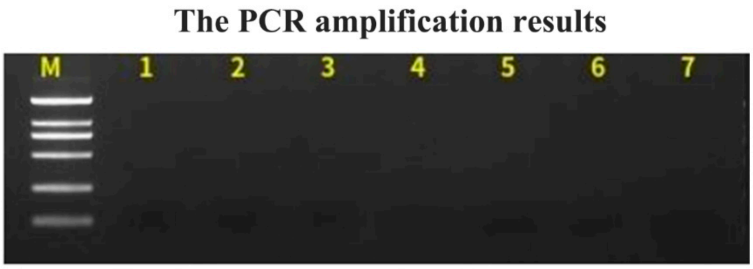

Figure S4. The PCR amplification results of AAD group mice's feces.

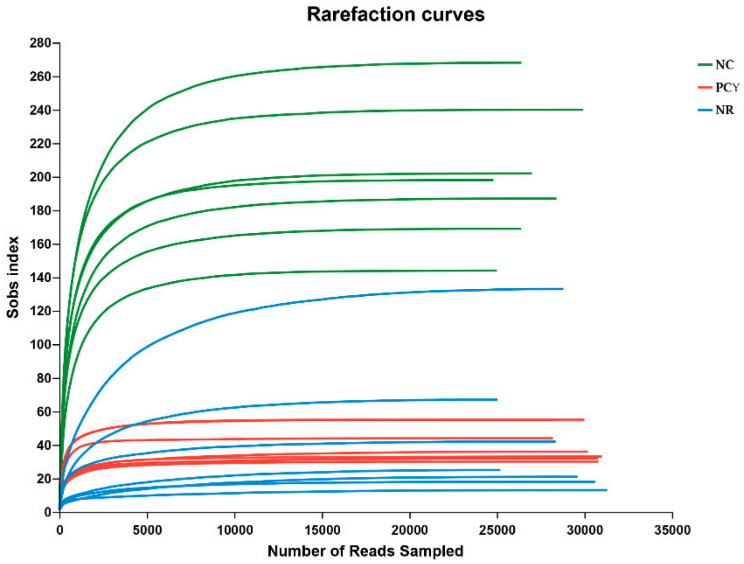

Figure S5. The Rarefaction curve.

Table S1. The α-diversity of microbial communities in mice.

	α -diversity		
**Sample/Estimators**	**Ace**	**Shannon**	**Simpson**
NC_1	187	3.746465	0.048439
NC_2	169	3.83913	0.04423
NC_3	268	4.285296	0.031314
NC_4	144	3.197569	0.091662
NC_5	202	4.078472	0.036249
NC_6	240	3.989338	0.068928
NC_7	198	4.016388	0.039845
NR_1	18	1.175987	0.340363
NR_2	25	0.724777	0.697876
NR_3	13	1.131619	0.392844
NR_4	67	1.611031	0.331836
NR_5	21	0.955724	0.438322
NR_6	42	1.752194	0.28794
NR_7	133	2.326103	0.170479
PCY_1	33	1.443235	0.409869
PCY_2	36	1.508711	0.385701
PCY_3	30	1.711096	0.294341
PCY_4	32	1.588236	0.340307
PCY_5	55	2.632956	0.1306
PCY_6	44	2.244156	0.19506
PCY_7	55	2.689457	0.122854

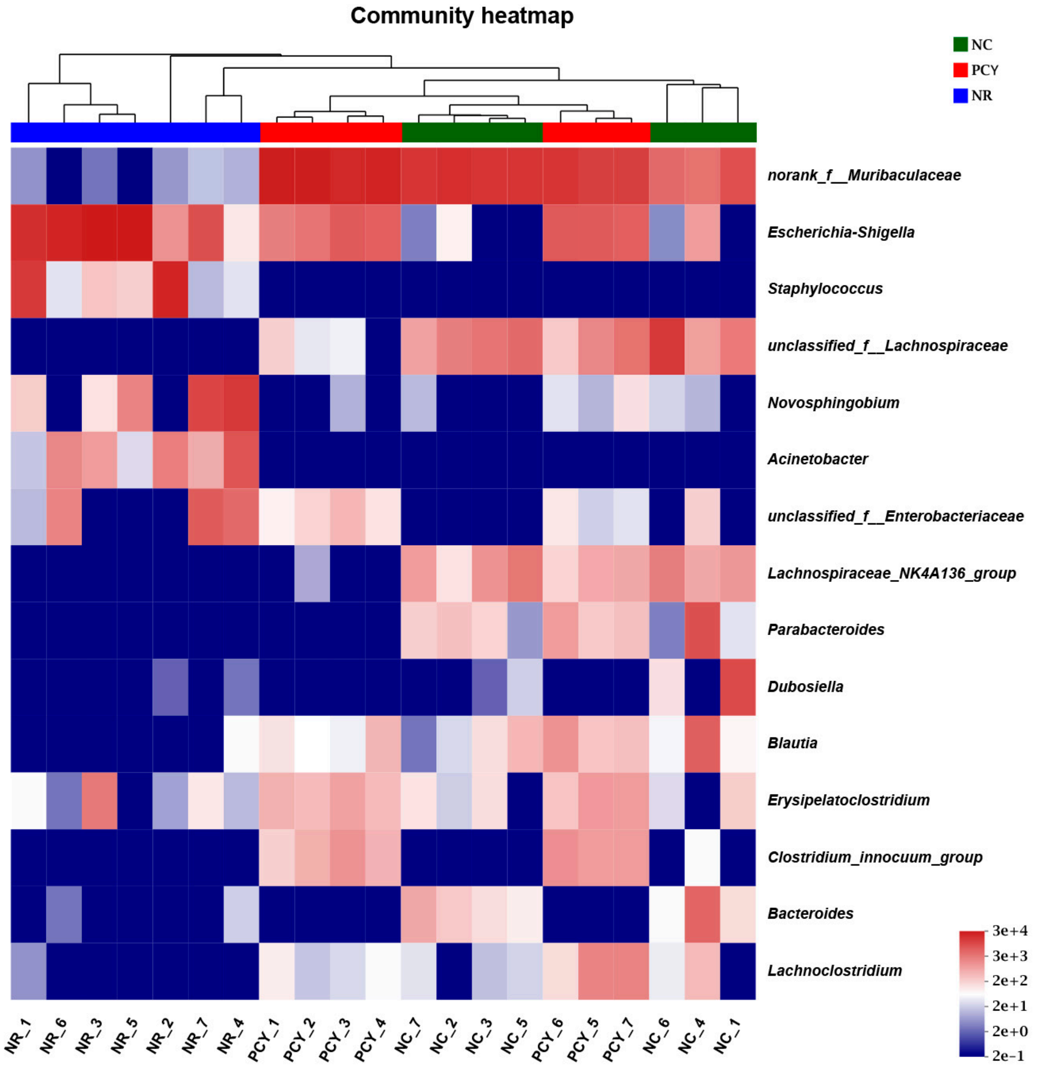

Figure S6. The heat map of microbial community at the genus level.

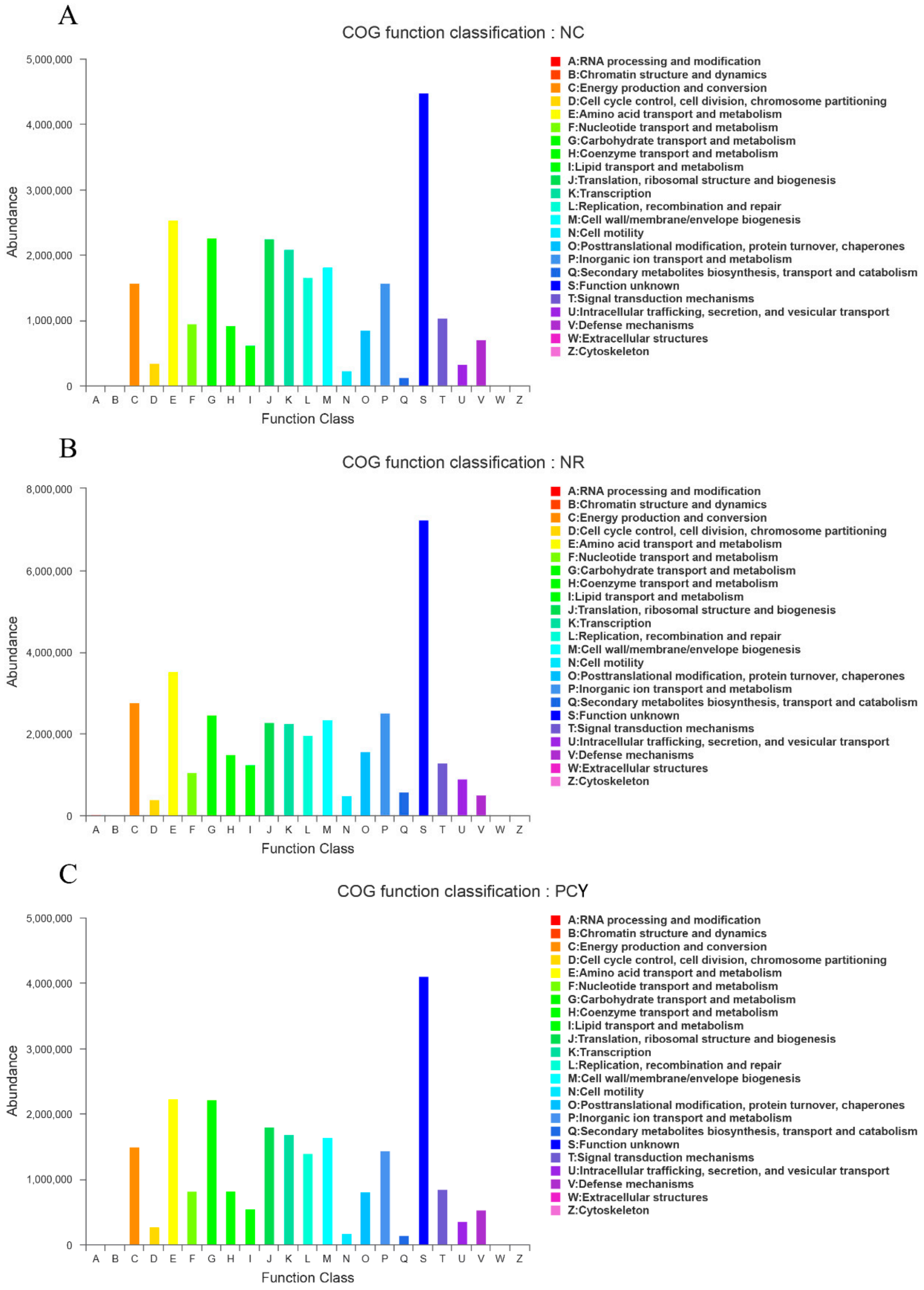

Figure S7. The COG function classification in three groups.

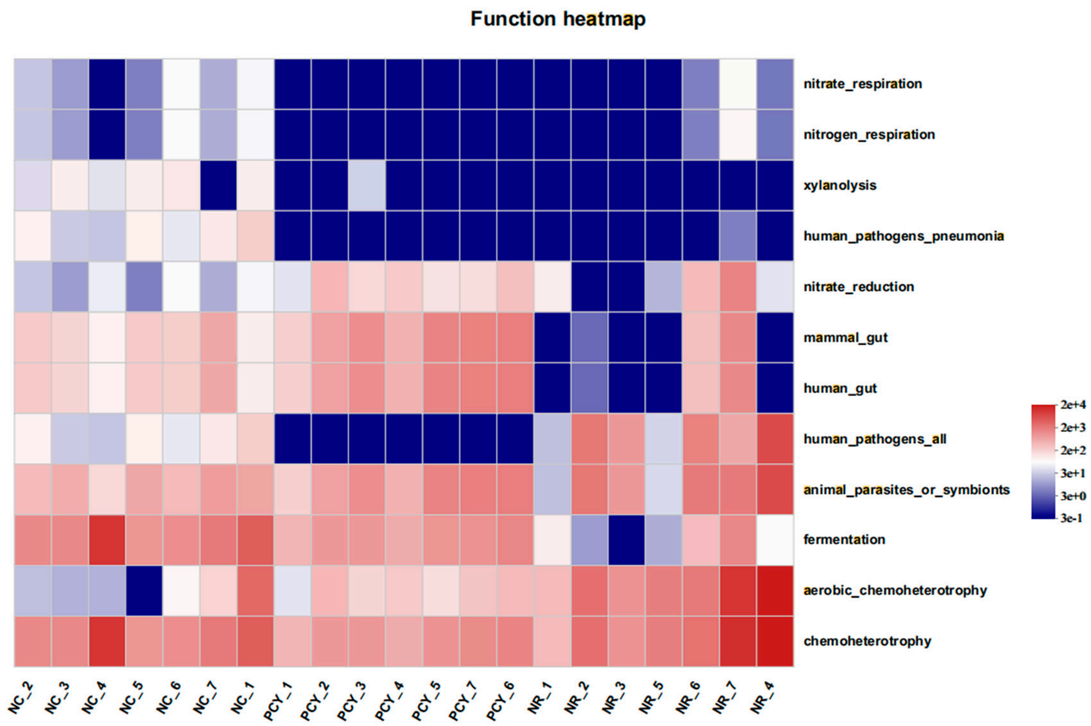

Figure S8. The correlation heat map of protein function prediction for the three groups

Table S2. The value of environmental factor VIF

The value of the environmental factor VIF before filtering								
Name	Acetic acid	Propanoic acid	Isobutyric acid	Butanoic acid	Isovaleric acid	Valeric acid	Isohexanoic acid	Hexanoic acid
VIF	6.289840491	28.33557156	16.87152043	36.06216208	9.777370928	69.95417405	3.498807404	4.0831299

The value of the environmental factor VIF after filtering					
Name	Acetic acid	Butanoic acid	Isovaleric acid	Isohexanoic acid	Hexanoic acid
VIF	2.699806075	3.40673885	4.917140524	1.801825538	2.842532451
